# Royal Jennerian Society

**Published:** 1808-10

**Authors:** John Ring, William Blair


### ROYAL JENNERIAN SOCIETY.

*Salisbury Square, August* 19, 1808.

THE Royal Jennerian Society for the extermination of the small-pox having received information that considerable alarm had been excited by reports of supposed failures in vaccination at Cambridge, requested the favour of Mr. Ring and Mr. Blair to proceed thither immediately, for the purpose of investigating those reports. In this request of the Society the above gentlemen readily acquiesced; and they have since laid before the Board of Directors a particular statement, of which the following is a general abstract.

*General Result of an Inquiry into the unfavourable Reports concerning Vaccination at Cambridge, by the Deputation of the Royal Jennerian Society, August 5th,* 1808.

The small-pox has been very prevalent and very fatal at Cambridge, for ten weeks past; during which period a considerable number of children, vaccinated at different times, have been much exposed to the infection of that disorder, which they have perfectly resisted.

In many instances, however, the small-pox was supposed to have occurred after vaccination; in some of which all doubts had been removed: but sixteen cases were deemed still deserving of investigation,

These cases may be divided into two classes: one, containing those in which there was no regular and complete vaccination; the other, those where the patients have not had the small-pox.

In some instances, vaccination did not take place in a satisfactory manner; yet these were reckoned among the failures in vaccination, by persons who were prejudiced against the practice.

In several examples there was only a festering of the arm; or the pock was essentially injured, and the security of the patient thereby diminished. In others, which fell under the observation of two respectable practitioners at Cambridge, and of the deputation who accompanied them, the chicken-pox was mistaken for the small-pox. In confirmation of this remark it is necessary to state, th echicken pox, as well as the small-pox, has for some time been, and still is, very prevalent at Cambridge.

In two cases, the patients were inoculated for the *small pox* in the manner of a *seton,* " by a bone-setter, who was was formerly a grocer," together with two of his own children; and a festering of the arms was produced. About a month ago, when the small pox appeared in these four cases, and proved fatal in one of them, he was applied to; then, and not till then, he pretended that he had not inoculated the children for the small pox, but for the cow pock!

When a child was inoculated for the cow-pock without effect, and afterwards successfully inoculated for the small-pox, this was represented to be *a failure in Vaccination!* and when another child, who had been vaccinated, was afterwards seized with a fever *without eruptions*, this also was represented to be *a failure in Vaccination!*—Having such prejudices to encounter, it is no wonder that the failures in this practice at Cambridge were supposed to be frequent; but, after a minute and careful inquiry into the circumstances which had excited so general an alarm, it did not appear that a single case of the small pox occurred there, after regular and complete vaccination.

In this inquiry the deputation of the Royal Jennerian Society were assisted by two eminent surgeons at Cambridge, who have had a considerable share in the practice of vaccination, one of them having vaccinated THREE THOUSAND PERSONS; and the notes of every case, submitted to their investigation, were read to the parents themselves, or the parties concerned, to prevent the possibility of mistake.

JOHN RING.
WILLIAM BLAIR.

---

A particular statement of the cases of supposed failure here alluded to, and the original minutes on which that statement is founded, may be inspected by any medical man, on application to Dr. Knowles, Resident Inoculator of the Society, at their Central House, No. 14, Salisbury Square.

*By Order of the Board of Directors.*
CHARLES MURRAY, Secretary.

CRITICAL

